# Testing Virulence of Different Species of Insect Associated Fungi against Yellow Mealworm (Coleoptera: Tenebrionidae) and Their Potential Growth Stimulation to Maize

**DOI:** 10.3390/plants10112498

**Published:** 2021-11-18

**Authors:** Eva Praprotnik, Jernej Lončar, Jaka Razinger

**Affiliations:** Plant Protection Department, Agricultural Institute of Slovenia, 1000 Ljubljana, Slovenia; jernej.loncar@kis.si (J.L.); jaka.razinger@kis.si (J.R.)

**Keywords:** entomopathogenic fungi, *Tenebrio molitor*, virulence, pathogenicity, growth stimulation, plant–microbe–pest interactions, rhizosphere competence

## Abstract

This paper investigates 71 isolates of two genera of entomopathogens, *Metarhizium* and *Beauveria*, and a biostimulative genus *Trichoderma*, for their ability to infect yellow mealworms (*Tenebrio molitor*) and to stimulate maize (*Zea mays*) growth. Fungal origin, host, and isolation methods were taken into account in virulence analysis as well. Isolates *Metarhizium brunneum* (1154) and *Beauveria bassiana* (2121) showed the highest mortality (100%) against *T. molitor*. High virulence seems to be associated with fungi isolated from wild adult mycosed insects, meadow habitats, and Lepidopteran hosts, but due to uneven sample distribution, we cannot draw firm conclusions. *Trichoderma atroviride* (2882) and *Trichoderma gamsii* (2883) increased shoot length, three *Metarhizium robertsii* isolates (2691, 2693, and 2688) increased root length and two *M. robertsii* isolates (2146 and 2794) increased plant dry weight. Considering both criteria, the isolate *M. robertsii* (2693) was the best as it caused the death of 73% *T. molitor* larvae and also significantly increased maize root length by 24.4%. The results warrant further studies with this isolate in a tri-trophic system.

## 1. Introduction

Entomopathogenic fungi are primarily known for their ability to parasitize insects and kill or severely harm them [1,2,3]. Fungi of the hypocrealean family Cordycipitaceae include important entomopathogens, of which certain species of *Metarhizium*, *Beauveria* and *Isaria* are most studied. The use of these typically facultative parasitic fungi as biopesticides is prevalent due to the wide range of target hosts and their ability to complete their life cycles also independently from insect hosts [4]. Entomopathogens can also colonize the rhizosphere and plant tissues as endophytes and act as plant growth promoters [5,6]. The occurrence of entomopathogenic endophytes is reported in more than 50 host plants, including cereals, legumes, oil and fiber crops, herbs, deciduous and coniferous trees, and others (reviewed in [7]). Their association with plants allows them to interact closely with insect herbivores in a tri-trophic system [8,9], ultimately impacting economic aspects, particularly in agriculture [10]. However, to design successful pest management strategies, it is necessary to fully understand the ecological role of implemented microbes.

Coating seeds with plant beneficial entomopathogens is a viable method for delivering microbes to germinating crops. It can be a cost-reducing alternative to soil inoculation, which requires large amounts of microbial inoculum, which could be an economic disadvantage if applied on a larger scale [11]. Seed coating can improve plant defenses by adding a certain concentration of beneficial organisms to the soil in the immediate vicinity of the germinated seed, which promotes seedling development and acts against plant pathogens or insect pests. For example, reducing wireworm pressure during the first three weeks of maize growth can significantly minimize crop loss [12,13]; therefore, a specific treatment that would enhance seed germination or speed up early-stage growth would be highly beneficial as it would give the plant an advantage to oppose soil pests. Secondly, the level of defense can be improved by direct insect interfering activities of entomopathogens. If present as endophytes and rhizosphere colonizers, they could directly protect plants at later physiological stages. Coating bean *Phaseolus vulgaris* L. seeds with *Beauveria bassiana* (Balsamo) Vuillemin and *Metarhizium robertsii* J.F.Bisch., Rehner and Humber significantly reduced the population of the spider mite *Tetranychus urticae* Koch while improving plant growth within five weeks after inoculation [14]. Similar effects were observed when maize and tobacco seeds were coated with *M. robertsii* [1,15] and white jute seeds with *B. bassiana* [16].

However, biotic and abiotic conditions, as well as genotypic and phenotypic plasticity of host plants and fungi, can significantly affect the insect associated fungi–host interactions [17]. Evolutionary theory supports the important role of grass endophytes in defense against herbivores in a mutualistic manner. However, this relationship is not fixed and may, under certain conditions, turn into a neutral or even antagonistic interaction [18]. As plants influence the chemical and nutritional properties of their rhizospheres, the entomopathogenic fungi living there are under strong selection pressure to utilize the specific rhizodeposits and might be subject to habitat selection rather than the presence/absence of an insect host [19,20]. Therefore, these factors should be considered, especially at tri (multi)-trophic levels when considering entomopathogens for commercial use.

Our question was whether the origin of the fungus, host, or the isolation method could affect the fungal virulence of the fungus and whether biostimulative properties are common among highly virulent fungal isolates. Therefore, we looked at 66 strains belonging to the entomopathogenic genera *Metarhizium* and *Beauveria*, as well as five from genus *Trichoderma*, primarily known as a biostimulative fungus [21,22], however, also a proven insects’ facultative pathogen [23,24,25,26] in order to evaluate their ability to infect yellow mealworms (*Tenebrio molitor* Linnaeus, 1758) and stimulate maize (*Zea mays* L.) growth. Mealworms are known for their susceptibility to entomopathogenic fungal infection and are a suitable test organism for assessing fungal virulence. Although they are mostly known as storage pests, their natural environment is dark and moist earth’s floor, mostly under rocks or in leaf-litter [27]. Maize served as a model plant because most isolates were isolated from maize fields or from the rhizosphere of wild Poaceae species growing in dry Karst meadows.

## 2. Results

### 2.1. Virulence Bioassay

Altogether 71 fungal isolates were analyzed for their virulence against *T. molitor* (Table 1). All isolates, with the exception of *Trichoderma atroviride* P.Karst. (number of strains tested: n = 2), *Trichoderma harzianum* Rifai (n = 1) and *Trichoderma gamsii* Samuels and Druzhin. (n = 1), showed pathogenicity against *T. molitor*. After 14 days *Metarhizium brunneum* Petch (n = 4) caused mortality ranging from 21.43 to 100.00%, while *M. robertsii* (n = 53) caused mortality ranging from 5.27 to 84.62%, and *Metarhizium guizhouense* Q.T. Chen and H.L. Guo (n = 3) caused mortality ranging from 3.70 to 32.14%. The isolates of *B. bassiana* (n = 5) caused mortality ranging from 53.33 to 100.00%. Isolates *B. bassiana* (2121) and *M. brunneum* (1154) had the highest Abbott’s corrected mortality after 7- and 14-days post inoculation. Three isolates of *B. bassiana*, two of *M. brunneum*, and two of *M. robertsii* caused mortality of at least 75% after 14 days.

### 2.2. Influence of Fungal Origin and Isolation Method on Mortality Rate

**Figure 1:**
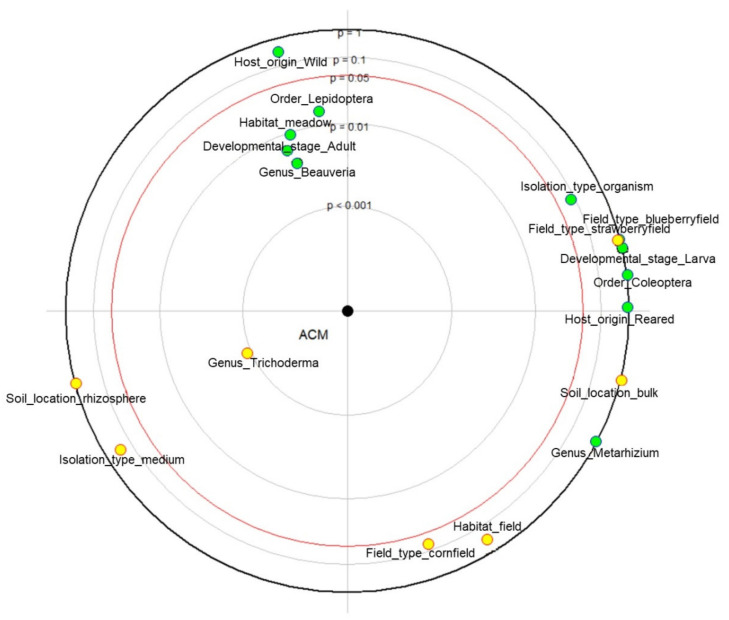
Correlation circle of predictor variables toward mortality rate (ACM). Green dots indicate positive correlation with the dependent variable and yellow dots indicate negative correlation with the dependent variable. Predictor variables within the red circle are significantly correlated with the dependent variable (*p* < 0.05).

For exploratory data analysis, we illustrated different parameters in correlation with ACM of 67 fungal isolates (Figure 1). The results indicate positive correlation between ACM and the genus *Beauveria*, adult Lepidoptera insect host, and meadows. Conversely, a negative correlation is shown between ACM and the genus *Trichoderma.*

We detected no significant difference in ACM on 14th day between isolates isolated from a wild host versus a reared host (F_1,50_ = 0.897, *p* = 0.348), from a live organism versus a selective medium (χ^2^(1) = 1.8712, *p* = 0.1713) and from bulk soil versus rhizosphere soil (F_1,56_ = 0.144, *p* = 0.706). On the other hand, we detected a significant difference in ACM on 14th day between isolates of different genera (χ^2^(2) = 18.423, *p* = 0.0001), isolates isolated from a meadow versus a field (F_1,57_ = 7.182, *p* = 0.0096), and marginally significant differences in ACM of isolates from an adult insect host versus larvae (χ^2^(1) = 4.0098, *p* = 0.0452) and from a Lepidoptera insect host versus a Coleoptera (χ^2^(1) = 4.1391, *p* = 0.0419).

### 2.3. Growth Stimulation Bioassay

Seventy-one fungal isolates were tested for stimulation of maize growth (Table 2). The average number of conidia per maize seed was 2.43 × 10^6^ ± 1.99 × 10^5^. There was a significant difference in the average number of conidia per maize seed between isolates of different genera (χ^2^(1) = 5.2406, *p* = 0. 0.0221) and between different habitats or origin of the isolate (χ^2^(4) = 21.219, *p* = 0.0002). Isolates that originated from maize fields had a higher number of conidia per maize seed (*p* ≤ 0.05) as opposed to isolates from meadows, soil, blueberry field, or insects. Furthermore, isolates of the genus *Metarhizium* had a higher number of conidia per maize seed as opposed to genus *Beauveria*.

In Chapalu variety, there was no significant effect of tested isolates on emergence success and total plant length (root + shoot length). ANOVA showed a significant prolongation of emergence time with two *M. robertsii* isolates (2698 and 2700) and one *T. atroviride* (2752). Root length was significantly reduced by two *M. robertsii* isolates (2243 and 2636). Shoot length was significantly reduced by *B. bassiana* (2299) but increased by *T. atroviride* (2882) and *T. gamsii* (2883). Plant dry weight was significantly reduced by two *B. bassiana* isolates (2299 and 2300) and *M. robertsii* (2011).

In Belokranjka variety, there was no significant effect of tested isolates on emergence success, shoot length, and total plant length (root + shoot length). ANOVA showed a significant prolongation of emergence time with *M. robertsii* (2154). Root length was significantly increased by three *M. robertsii* isolates (2691, 2693, and 2688) and plant dry weight was significantly increased by two *M. robertsii* isolates (2146 and 2794).

### 2.4. Enhancement of Nutrient Utilization by Fungi in Maize

Thirty fungal isolates were further tested for growth stimulation of maize (Chapalu variety only) in sand with or without fertilizers (General Hydroponics, Flora Series^®^) (Table 3). In the absence of fertilizer, there was no significant effect of the tested isolates on emergence success and total plant length. However, ANOVA showed a significant prolongation of emergence time with *M. guizhouense* (2010) and a significant reduction in root length with *B. bassiana* (2299) and *M. robertsii* (2148). Shoot length was significantly reduced by *M. robertsii* (2642) but increased by *M. robertsii* (2011). Plant dry weight was significantly increased by *M. robertsii* (2216) in unfertilized sand.

There was no significant effect of tested isolates on emergence success, root length, total plant length, and dry weight in the presence of fertilizer. ANOVA showed a significant prolongation of emergence time of maize treated with *M. brunneum* (2703), *M. robertsii* (2009), and *M. guizhouense* (2010). Shoot length was significantly increased with *M. robertsii* (2011) in fertilized sand.

When all data were analyzed together, fertilization caused an average increase in shoot length of 32.3% (t(74) = 13.13, *p* = < 0.0001) and an increase in total plant length of 13.2% (t(74) = 6.47, *p* = < 0.0001) compared to unfertilized plants. Fertilizer itself had no significant effect of on emergence success, emergence time, root length and plant dry weight.

Two-way ANOVA was performed to determine if fungal isolates altered the growth parameters of maize in fertilized vs. unfertilized sand compared to untreated maize. Dunnett’s multiple comparison test showed a prolonged emergence time in fertilized sand when treated with *B. bassiana* (2009). The isolate *M. guizhouense* (2010) prolonged emergence time in fertilized sand as well as in unfertilized sand. *M. robertsii* (2631) significantly increased root length in unfertilized sand and *M. robertsii* (2632) in fertilized sand. On the other hand, *B. bassiana* (2299) and *M. robertsii* (2148) significantly reduced root length in unfertilized sand. Two isolates of *M. brunneum* (1868 and 1154) and *B. bassiana* (2121) increased dry weight in fertilized sand.

Fungal treatment had a significant effect on emergence time in two out of eight experiments, on root length in three out of eight experiments, on shoot length in four out of eight experiments, and on plant dry weight in one out of eight experiments. However, no significant effect of fungi on emergence success was observed.

Fertilization significantly affected emergence time and plant dry weight in one out of eight experiments, root length in two out of eight experiments, and in all experiments the presence of fertilizer significantly affected shoot length. No significant effect of fertilizer on emergence success was observed.

## 3. Discussion

A total of 71 fungal isolates were obtained mainly from soil samples, by using the *Galleria–Tenebrio* bait method, but also using selective media and mycosed insects found in different agroecosystems. Overall, the most frequently isolated representatives were *M. robertsii* (i.e., 75% of isolates). Therefore, it is possible that the isolation techniques favor this species. However, Sharma et al. [28] also used the *Galleria-Tenebrio* bait method, where twice as many *B. bassiana* than *M. robertsii* were isolated. Moreover, Medo and Cagáň [29] used the *Galleria* bait method to isolate fungi, but the predominant species was *B. bassiana* and no *M. robertsii* was isolated.

In the present study, only *M. robertsii* and *M. guizhouense* were isolated with *Tenebrio* as bait, while with *Galleria* as bait approximately half of the isolates were *B. bassiana* and the other half belonged to the genus *Metarhizium*. There are some reports where *B. bassiana* was recovered more frequently when *Galleria* was used as bait, while *Tenebrio* bait resulted in more frequent isolation of *Metarhizium* species [28,30,31]. Therefore, to obtain more representative and less biased information about the entomopathogenic fungal community in an agroecosystem, it is recommended to increase the number of arthropod species used as bait.

A total of 71 isolates were tested for virulence against *T. molitor* and maize growth stimulation. The isolates differed significantly in their degree of virulence. The most virulent isolates were those obtained from lepidopteran insect hosts and from mycosed wild adult coleopterans. One would expect higher virulence from isolates derived from *T. molitor* baits, which is the same species as the model insect used in our bioassays, but this was not the case in our study. The positive controls (Actara, Force) and the commercial bioinsecticides (Mycotal, Met52) showed very low mortality after 14 days: Mycotal, Force, and Met52 around 10% ACM or less, and Actara less than 50% ACM. Mycotal and Met52 are both biopesticides primarily intended for whitefly and thrips control (Met52 also fungus gnats and mites), but have also been tested for Coleoptera [32,33]. Force and Actara are, among others, used to control coleopteran pests. Although mortality was low 14 days after Actara treatments, it is worth noting that mealworms were ecologically dead (i.e., insects were lethargic, spasming, no longer feeding) a few days after treatment.

Although our analysis suggests a higher correlation of *B. bassiana* with mortality, one cannot conclude that one species is more pathogenic than the others. The level of virulence often varies within species and even clades, as shown by the phylogenetic analyses of Medo et al. [34] and Lopes et al. [35]. Moreover, the seven most virulent isolates in our study belong to three different species. The origin of soil samples and their chemical and physical properties can have significant effects on the presence, abundance, and pathogenicity of insect-associated entomopathogens. For example, the infection rate of pupae of the Mediterranean fruit fly, *Ceratitis capitata* (Wiedemann) was higher with fungi isolated from soils with a sandy texture and high organic matter content [36] and in soils with a water potential of –0.1 MPa [37].

Our study also suggests a stronger association of *B. bassiana* with meadows. Higher abundance and diversity of *B. bassiana* in more semi-natural habitats and less physically disturbed soils has also been observed in other studies and is likely the result of many biotic and abiotic factors, such as increased humidity, reduced ultra-violet radiation, and temperature, reduced agricultural activities (e.g., tillage or fungicide use), higher insect diversity, etc. [38,39].

*Trichoderma* species are important biological control agents due to their antagonistic properties against various pathogenic fungi. Some species are capable of colonizing plants, including maize, in addition to increasing photosynthetic rate [40], root and shoot growth, plant biomass [41], and enhancing the immune system of plants [42]. However, there are also a few reports on the entomopathogenic properties of *Trichoderma*, where direct damage to insect pests has been observed. *Trichoderma viride* Pers. derived chitinases have effectively degraded the chitinous vital structures of *Bombyx mori* (Linnaeus, 1758) larvae [26], *Trichoderma koningiopsis* Samuels, Carm. Suárez and H.C.Evans have shown significant entomotoxicity against *Delia radicum* (L.) pupae in the soil environment, and *T. atroviride* against *D. radicum* eggs in in vitro tests [25], while *T. harzianum* caused up to 80% larval mortality against the Egyptian cotton leafworm *Spodoptera littoralis* (Boisduval, 1833) [23] and up to 100% mortality of *T. molitor* larvae [24]. In contrast, the *Trichoderma* isolates tested in this study showed little or no pathogenicity against *T. molitor*. In terms of stimulation of maize growth, shoot length was significantly increased by 25.8% by *T. atroviride* (2882) and 22.5% by *T. gamsii* (2883). However, significant prolongation of emergence time was observed with *T. atroviride* (2752). Ousley et al. [43] also reported no significant growth-promoting or even inhibitory properties of *Trichoderma*, especially in relation to germination rate. *Metarhizium robertsii* isolates (2698, 2700 and 2154) also significantly prolonged the emergence time. Razinger et al. [44] and Kuzhuppillymyal-Prabhakarankutty et al. [45] also reported a lower germination rate of maize seed; this could be a consequence of the method by which the conidia were applied to the seeds, namely by using carboxymethyl cellulose or methylcellulose. In our case, the maize seeds were soaked in a suspension of fungal conidia using only 0.1% Tween 80 to overcome the difficulties with the hydrophobic properties of the conidia of the fungal species under study and to allow adequate adhesion of the conidia to the maize kernels. Therefore, the method of conidia attachment to the seeds used may not be the (only) reason for the inhibition of germination and emergence; more likely the reason lies in the fungi tested. It should be noted that entomopathogenicity may have evolved later, especially within the Clavicipitaceae, meaning that their ancestors used plants or plant debris as a food source [46]. This could explain the inhibitory effect of entomopathogenic fungi, as their metabolites, i.e., destruxins, might also be toxic for plants [47].

In general, there are very few studies observing the emergence speed of plant seeds treated with entomopathogens [48,49]. The focus of most research is more prone to study germination rate rather than emergence time. However, rapid and reliable emergence is of particular importance to maize seed growers, especially in temperate regions, where maize is usually planted in spring in soil with suboptimal temperatures for emergence [50]. Rapid emergence also shortens the time plants are exposed to (soil) pests and reduces weed infestation [51,52].

*Metarhizium* and *Beauveria* species as typical entomopathogens were also tested for their growth stimulation properties to maize. *Beauveria bassiana* isolate (2299) significantly reduced shoot length and plant dry weight (isolates 2299 and 2300) in the variety Chapalu. Rivas-Franco et al. [53] also noticed a reduction in root and shoot dry weight in maize seeds treated with *B. bassiana*. However, Kuzhuppillymyal-Prabhakarankutty et al. [45] observed higher plant dry and fresh weight as well as better performance of coated maize exposed to drought. In addition, Russo et al. [54] detected positive effects on all yield characteristics, seed germination, and measured growth parameters when maize was inoculated by a leaf spraying technique. Tall and Meyling [55] reported increased root and shoot biomass in maize treated with *B. bassiana* and grown in nutrient-rich soil. However, when nutrient availability was low, they observed reduced plant growth compared to the control, which may indicate that fungi act as potential resource sink. Our results showed no significant growth stimulation of maize treated with *B. bassiana* growing in sand with added fertilizers. However, in the absence of fertilizers, *B. bassiana* (2299) and *M. robertsii* (2148) showed a significant decrease in root length and *M. robertsii* (2642) also showed a decrease in shoot length, which could indicate the uptake of nutrients by the fungi in an environment where resources are scarce.

*Metarhizium robertsii* isolates significantly reduced root length (isolates 2243 and 2636) and plant dry weight (isolate 2011) in the Chapalu variety. In contrast, other isolates of the same species significantly increased root length (isolates 2691, 2693, and 2688) and plant dry weight (isolates 2146 and 2794) in the Belokranjka variety. The effects of coating maize seeds with *Metarhizium* are often beneficial. Razinger et al. [44] reported a significant increase in fresh weight of maize by coating seeds with *M. robertsii*, but no effect on plant length, whereas colonized maize plants of Ahmad et al. [1] were greater in length and shoot biomass. Kabaluk and Ericsson [56] treated maize seeds with *Metarhizium anisopliae* (Metschn.) Sorokīn conidia, which resulted in increased stand density and plant fresh weight in a wireworm-infected field. However, their laboratory experiments showed that treating maize with 3.8 × 10^8^ conidia per seed actually reduced seed germination and root growth, indicating the possibility of a potential limit of conidia per seed at which seed viability is not at risk.

## 4. Materials and Methods

### 4.1. Isolation of Fungi

Entomopathogenic fungi were isolated either from naturally present sporulating insect cadavers, or from soil samples using *Galleria mellonella* (Linnaeus, 1758) and *T. molitor* larvae as bait [57], or from serially diluted soil suspensions plated on semi-selective media as described by Cooke [58] and Williams et al. [59]. In the latter two cases, soil samples were obtained from maize fields (mainly bulk soil) or from Karst extensive hay meadows, accommodating a high diversity of Poaceae species (soil from the Poaceae rhizosphere). Sampling sites and host/medium characteristics are summarized in Table 1. A Nikon (SMZ800, Nikon Corp., Melville, NY, USA) binocular was used to identify sporulating structures formed by fungi on cadavers that were placed in droplets of sterile water to generate spore suspensions. Aliquots of the suspension were moved over the surface of potato dextrose agar supplemented with bacteria suppressing antibiotics (streptomycin and penicillin) to generate single spore cultures. The isolates obtained were identified on the basis of morphological characters seen on the insect cadavers or in pure culture or through DNA barcoding according to Razinger et al. [60]. In brief, molecular barcode sequences of the intron-rich part of the elongation factor 1-alpha (*tef*) were obtained by adopting the strategies described by Bischoff et al. [61] but using the EF2 primer of O’Donnell et al. [62]. The 50 μL reaction mixture for PCR consisted of 5 μL of Taq PCR buffer with (NH4)2SO4 (Fermentas, Waltham, MA, USA), 2 mM MgCl2, 0.2 mM dNTP (Promega, Madison, WI, USA), 0.5 mM of each of the primers, 1 unit of native Taq polymerase (Fermentas, Waltham, MA, USA) and 1 μL of genomic DNA. In PCR, we used an initial denaturation step at 94 °C for 3 min, 5 cycles of 94 °C for 60 s (denaturation), 56 °C for 45 s (annealing), 72 °C for 60 s (elongation), and 35 cycles as described before but with an annealing temperature of 53 °C, and a final extension at 72 °C for 8 min. Sequencing reactions were performed at the Macrogen Europe sequencing facility (Amsterdam, The Netherlands) in both directions by using the same primers as used in PCR. The data were inspected and edited with the aid of the software program BioEdit v7.2.0 [63]. Representative sequences were deposited at NCBI database.

### 4.2. Fungal Virulence toward Tenebrio molitor

Single-dose virulence testing was performed on larvae of mealworms *T. molitor*, reared at the Agricultural Institute of Slovenia. Fungal strains were subcultured on Potato Dextrose agar (PDA; Biolife, Italy) and incubated in an incubation chamber (IPP 500, Memmert) at 22 °C for 14 days or longer to obtain the required amount of sporulating structures. Spores were washed-off by pipetting approximately 10–15 mL of sterile 0.1% Tween 80 solution onto the top of cultures and scraping colonies with a Drigalski spatel. The obtained suspensions were collected into sterile 50 mL Falcon tubes. Haemocytometer (Bürker-Türk, BRAND GMBH + CO. KG, Wertheim, Germany) counting was used to adjust obtained suspensions to a concentration of 1 × 10^8^ conidia ml^−1^ [44]. The viability of conidia was determined by counting germinated conidia after 24 h of incubation of the diluted suspension sample.

Thirty larvae per strain were immersed in 1 × 10^8^ mL^−1^ conidial suspension for 15 s, with a slight stirring. Two commercial insecticides were used as positive controls: 0.1% tap-water dilution of Actara 25 WG (Syngenta, Switzerland; active ingredient Thiamethoxam, 25% *w*/*w*) and 0.1% Force 1.5G (Syngenta, Switzerland; a.i. Tefluthrin, 0.15% *w*/*w*). In addition, two commercial bioinsecticides were used as reference biocontrol agents: 0.1% Mycotal (Koppert, Netherlands; a.i. *Lecanicillium muscarium* (Petch) Zare and W.Gams Ve6) and 1% Met52 EC (Novozymes, France; a.i. *M. brunneum* strain F52). Sterile 0.1% Tween 80 was used as a negative control. Mealworms were afterwards transferred into a petri dish (each strain to a separate Petri dish) and allowed to dry under a laminar flow hood for 20–30 min. Each mealworm was placed in its own well in a six-well plate with a few pieces of oatmeal as food. Five replicates of six-well plates were made per strain (n = 30 per strain). Treated mealworms were kept in a loosely closed cardboard box in an incubation chamber for 2 weeks set to 75% r.h., 21 °C and 14:10 h (light:dark) regime. The number of dead or immobile larvae was checked every 3 days. Dead larvae were incubated at room temperature on water agar to confirm infection by the fungi. For further information on the virulence bioassay see Supplementary Materials Figures S1 and S2.

### 4.3. Maize Growth Biostimulation Tests

#### 4.3.1. Maize Seed Treatment

The fungal suspensions for the growth stimulation trials were prepared as described above. Maize seeds were soaked in the suspension or in sterile 0.1% Tween 80 (control treatment) and placed on an orbital shaker for 1 h and 15 min at 200 RPM. The seeds were then placed on filter paper and dried in a laminar flow hood for 1 h.

For each experiment, the number of conidia of each fungal strain per maize seed was evaluated. Three ml of 0.1% Tween 80 was added to 10 inoculated maize seeds in a Falcon tube (Deltalab, Barcelona, Spain) and vortexed for 10 s at 3000 rpm. The Falcon tube was left on an orbital shaker for 30 min at 600 rpm and afterwards vortexed again for 10 s at 3000 rpm. The number of conidia was determined using a hemocytometer [64].

#### 4.3.2. Growth Stimulation Bioassay

Two maize varieties, namely Chapalu (Saatzucht Gleisdorf, Austria) and Belokranjka (Organic farm Župnca, Slovenia), were used for the growth stimulation assays. The experiments with Chapalu variety were conducted with 5 seeds and 3 replicates and with Belokranjka variety with 10 seeds and 3 replicates. Seventy-one fungal isolates were tested for potential growth stimulation of maize in (i) twice autoclaved commercial planting substrate (Potgrond H, Klasmann, Germany).

Coated seeds were planted in 12 L plastic pots containing the substrates and kept in an incubation chamber at 22 °C/20 °C day/night temperature with a photoperiod of 14:10 h (light:dark) and 70–75% r.h. The number of emerged sprouts was counted every day until the end of seedling emergence. Three weeks after planting, growth parameters such as root length, shoot length and plant dry weight were measured on the harvested maize plants. For obtaining the dry weight, the substrate was carefully washed from the roots and all plants from one pot were placed in a paper bag, dried at 60 °C for 48 h, and weighed (BP301S, Sartorius).

#### 4.3.3. Fungal Nutrient Utilization Enhancement in Maize

Thirty fungal isolates were further tested for their potential enhancement of nutrient utilization in Chapalu variety only. Tests were performed in (ii) non-autoclaved sand and (iii) non-autoclaved sand with mineral fertilizers FloraMicro:FloraGro:FloraBlooom (General Hydroponics, Flora Series^®^, Europe) added to the sand on the 7th and 14th day of the experiment in the ratio FloraMicro:FloraGro:FloraBlooom = 2:1:1 mL per 3.79 L of water on day 7 and 4:5:1 mL per 3.79 L of water on day 14.

Coated seeds were planted in 0.25 L plastic pots with fertilized or unfertilized sand. The growth conditions and evaluation parameters were the same as in the ‘Growth stimulation bioassay’.

### 4.4. Data Analysis

The time-based larval mortality was analyzed using Kaplan–Meier survival analysis and its significance was analyzed using the log-rank (Mantel–Cox) test. When multiple survival curves were compared, the significance threshold was corrected using the Bonferroni method [65]. Survival analysis and calculation of median lethal time (LT50) were performed using GraphPad Prism 5.00 (GraphPad Software, Inc., La Jolla, CA, USA). Additionally, Abbott’s corrected mortality (ACM) was calculated to eliminate the effect of natural or unexplained mortality of the negative control group [66].

Focused principal component analysis (FPCA) was implemented for a more accurate interpretation of correlation of predictor variables, in our case fungal origin, habitat characteristics, and isolation method, toward mortality rate (ACM) using the packages “psy” [67] and “dummies” [68] in R 3.6.1 [69]. Selected parameters were as follows: genus of the isolates, habitat type (field vs. meadow), soil sample location (bulk vs. rhizosphere), field type, isolation method/type (insect host vs. selective medium), insect host order, and their origin (wild vs. reared) and developmental stage (adult vs. larva). The significance of the analysis was tested using the non-parametric Kruskal–Wallis test, followed by Dunn’s post hoc test, where the *p*-value was adjusted using the Benjamini–Hochberg (BH) correction. Normally distributed data were tested using the one-way ANOVA, followed by a post hoc Tukey HSD test. For this purpose, packages “dplyr” [70] and “rstatix” [71] were used.

All growth stimulation data were subjected to one-way ANOVA followed by a Bonferroni–Holm multiple comparisons test. For experiments where fertilizer was one of the parameters, also two-way ANOVA, followed by Dunnett’s multiple comparison test, was used in order to compare the effect of substrate (fertilized vs. unfertilized) and fungal isolates on the growth parameters of Chapalu maize. The analyses were carried out using GraphPad Prism software.

## 5. Conclusions

The aim of this study was to find the ideal fungal isolate that would combine two important characteristics of entomopathogenic and biostimulative fungi, namely the ability to infect insect pests and promote plant growth, and to test whether fungal virulence depends on the source of the isolate(s). The isolates *M. brunneum* (1154) and *B. bassiana* (2121) showed the highest mortality (100%) against *T. molitor*. High virulence was observed in isolates from wild adult mycosed insects, meadow habitats, and Lepidopteran hosts, but due to the uneven distribution of samples, we cannot draw any conclusive inferences. *Trichoderma atroviride* (2882) and *T. gamsii* (2883) showed the greatest promotion of plant growth, followed by two *M. robertsii* isolates (2693 and 2794). Even though we did not find the super fungus, *M. robertsii* (2693) came closest to meet our requirements. Maize seeds inoculated with this isolate showed a positive effect on all measured growth and emergence parameters while causing the death of 73% of *T. molitor* larvae. Therefore, it would be beneficial to test this isolate in a tri-trophic system that also includes a pest organism, e.g., wireworms, to determine its potential effect on maize stand density and/or yield increase.

## Figures and Tables

**Table 1:** Origin and virulence of selected fungal isolates against larvae of *Tenebrio molitor*. ACM–Abbott’s corrected mortality 7 and 14 days after inoculation; LT50–Median lethal time of *T. molitor* in days; Green fill indicates most virulent isolates with ACM 14 days after inoculation >75%, yellow fill indicates moderately virulent isolates with ACM 14 days after inoculation between 50% and 75%; * Asterisk indicates significance for survival curve analysis; Italic font indicates unreliable ACM results due to high control mortality (sterile 0.1% Tween 80).

Isolate	Taxon	Habitat or Origin	Isolation Type/ Host Organism	Host Developmental Stage	Host Origin	ACM 7.00 (%)	ACM 14.00 (%)	LT_50_ (d)
**1154**	*MB*	soil	*Galleria mellonella*	larvae	reared	61.54	100.00	6.00 *
**1868**	*MB*	meadow	*Agriotes* sp.	adult	wild	26.92	86.96	8.00 *
**2121**	*BB*	cauliflower field	Curculionidae	adult	wild	65.38	100.00	6.00 *
**2631**	*MR*	maize field	*Tenebrio molitor*	larvae	reared	10.34	52.00	12.5 *
**2632**	*MR*	maize field	*Tenebrio molitor*	larvae	reared	10.34	32.00	
**2245**	*MR*	maize field	*Tenebrio molitor*	larvae	reared	6.90	28.00	
**2246**	*MR*	maize field	*Tenebrio molitor*	larvae	reared	0.00	20.00	
**2215**	*MR*	maize field	*Tenebrio molitor*	larvae	reared	11.11	37.50	14.00
**2216**	*MR*	maize field	*Tenebrio molitor*	larvae	reared	7.41	54.17	10.00 *
**2299**	*BB*	meadow	*Galleria mellonella*	larvae	reared	18.52	54.17	10.00 *
**2300**	*BB*	meadow	*Galleria mellonella*	larvae	reared	33.33	79.17	10.00 *
**2635**	*MR*	maize field	*Tenebrio molitor*	larvae	reared	21.43	65.38	11.50 *
**2637**	*MR*	maize field	*Tenebrio molitor*	larvae	reared	17.86	84.62	11.00 *
**2641**	*MR*	maize field	*Tenebrio molitor*	larvae	reared	21.43	65.38	11.00 *
**2697**	*ND*	maize field	*Tenebrio molitor*	larvae	reared	*−18.18*	*−23.08*	
**2298**	*BB*	meadow	*Galleria mellonella*	larvae	reared	*42.11*	*88.24*	6.00 *
**2243**	*MR*	maize field	*Tenebrio molitor*	larvae	reared	0.00	37.50	14.00
**2151**	*MR*	maize field	*Tenebrio molitor*	larvae	reared	*−18.18*	*20.00*	13.00
**2703**	*MB*	soil	ND	ND	ND	0.00	21.43	*
**2009**	*MR*	soil	selective medium	-	-	10.34	32.14	14.00 *
**2010**	*MG*	soil	selective medium	-	-	3.45	32.14	*
**2011**	*MR*	soil	*Galleria mellonella*	larvae	reared	16.67	31.03	
**2686**	*MR*	maize field	*Tenebrio molitor*	larvae	reared	7.41	43.48	13.00 *
**2687**	*MR*	maize field	*Tenebrio molitor*	larvae	reared	11.11	56.52	11.00 *
**2690**	*MB*	soil	ND	ND	ND	7.41	47.83	12.00 *
**2692**	*MR*	maize field	*Diabrotica v. virgifera*	adult	wild	7.41	39.13	14.00 *
**2152**	*MR*	maize field	*Tenebrio molitor*	larvae	reared	−3.45	26.92	*
**2146**	*MR*	maize field	*Tenebrio molitor*	larvae	reared	20.00	54.17	11.00 *
**2147**	*MR*	maize field	*Tenebrio molitor*	larvae	reared	23.33	58.33	11.00 *
**2251**	*MR*	maize field	*Tenebrio molitor*	larvae	reared	16.67	75.00	11.00 *
**2789**	*MR*	maize field	*Diabrotica v. virgifera*	larvae	wild	16.67	41.67	13.00 *
**2793**	*MR*	maize field	selective medium	-	-	20.00	70.83	11.00 *
**2794**	*MR*	maize field	selective medium	-	-	10.00	20.83	
**2795**	*MR*	maize field	selective medium	-	-	36.67	66.67	11.00 *
**2645**	*MR*	maize field	*Tenebrio molitor*	larvae	reared	3.33	26.92	
**2691**	*MR*	blueberry field	*Tenebrio molitor*	larvae	reared	6.67	53.85	11.00 *
**2693**	*MR*	blueberry field	*Tenebrio molitor*	larvae	reared	6.67	73.08	11.00 *
**2790**	*MR*	maize field	*Diabrotica v. virgifera*	larvae	wild	0	19.23	14.00
**2634**	*MR*	maize field	*Tenebrio molitor*	larvae	reared	10.34	8.33	
**2214**	*MR*	maize field	*Tenebrio molitor*	larvae	reared	10.34	33.33	14.00
**2702**	*MR*	maize field	*Tenebrio molitor*	larvae	reared	0	8.33	
**2791**	*MR*	maize field	selective medium	-	-	37.93	62.50	12.00 *
**2250**	*MG*	maize field	*Tenebrio molitor*	larvae	reared	3.33	3.70	
**2685**	*MR*	maize field	*Tenebrio molitor*	larvae	reared	3.33	14.81	
**2640**	*MR*	maize field	*Tenebrio molitor*	larvae	reared	17.24	29.63	
**2694**	*MR*	strawberry field	*Tenebrio molitor*	larvae	reared	−3.45	25.93	
**2695**	*MR*	strawberry field	*Tenebrio molitor*	larvae	reared	6.90	22.22	
**2788**	*MR*	maize field	*Diabrotica v. virgifera*	larvae	wild	10.34	29.63	
**2792**	*MR*	maize field	selective medium	-	-	3.45	29.63	
**2796**	*MR*	maize field	selective medium	-	-	3.45	22.22	
**2688**	*MR*	maize field	*Tenebrio molitor*	larvae	reared	3.33	5.27	*
**2154**	*MR*	maize field	*Tenebrio molitor*	larvae	reared	3.57	*−23.81*	
**2153**	*MR*	maize field	*Tenebrio molitor*	larvae	reared	3.33	23.33	*
**2217**	*MR*	maize field	*Tenebrio molitor*	larvae	reared	3.33	10.00	
**2698**	*MR*	basil leaf	unknown larva	larvae	wild	3.33	13.33	
**2699**	*MR*	blueberry field	*Tenebrio molitor*	larvae	reared	3.33	10.00	
**2700**	*MR*	maize field	*Tenebrio molitor*	larvae	reared	10	20.00	
**2701**	*MR*	maize field	*Tenebrio molitor*	larvae	reared	3.33	13.33	
**2239**	*MR*	maize field	*Tenebrio molitor*	larvae	reared	0	20.00	
**2704**	*BB*	meadow	unknown larva	larvae	wild	0	53.33	14.00 *
**2247**	*MG*	maize field	*Tenebrio molitor*	larvae	reared	3.33	13.33	
**2752**	*TA*	decaying corn ear	natural substratum	-	-	0	0.00	
**2815**	*TB*	maize field	selective medium	-	-	0	3.33	
**2878**	*TH*	maize field	selective medium	-	-	0	0.00	
**2882**	*TA*	maize field	selective medium	-	-	0	0.00	
**2883**	*TG*	maize field	selective medium	-	-	0	0.00	
**2150**	*MR*	maize field	*Galleria mellonella*	larvae	reared	16.67	46.67	*
**2240**	*MR*	maize field	*Tenebrio molitor*	larvae	reared	23.33	50.00	14.00 *
**2148**	*MR*	maize field	*Galleria mellonella*	larvae	reared	23.33	53.33	14.00 *
**2636**	*MR*	maize field	*Tenebrio molitor*	larvae	reared	16.67	63.33	12.50 *
**2642**	*MR*	maize field	*Tenebrio molitor*	larvae	reared	3.33	23.33	*
**Actara 25 WG**	-	-	-	-	-	11.86	47.37	13.50 *
**Mycotal**	*LM*	-	-	-	-	−1.69	−3.51	
**Force**	-	-	-	-	-	1.69	1.75	
**Met52 EC**	*MB*	-	-	-	-	2.54	11.18	

Note: Actara 25 WG–insecticide based on the active ingredient Thiamethoxam (25% *w*/*w*); Mycotal–biological insecticide based on the active ingredient *L. muscarium* strain Ve6; Force 1.5G–insecticide based on the active ingredient Tefluthrin (0.15% *w*/*w*); Met52 EC–biological insecticide based on the active ingredient *M. brunneum* strain F52. *MB: Metarhizium brunneum*; *MR: Metarhizium robertsii*; *MG: Metarhizium guizhouense*; *BB: Beauveria bassiana*; *LM: Lecanicillium muscarium*; *TA: Trichoderma atroviride*; *TB: Trichoderma brevicompactum*; *TG: Trichoderma gamsii*; *TH: Trichoderma harzianum*; *ND*: No data.

**Table 2:** Growth stimulating effects of maize treated with selected fungal isolates and grown in twice autoclaved substrate for 21 days. Data presented are the mean values ± SE (n = 15 for Chapalu variety (3 replicates with 5 seeds each) and n = 30 for Belokranjka variety (3 replicates with 10 seeds each). Green fill indicates isolates with significant growth promoting properties, red fill indicates isolates with growth inhibitory properties and grey fill indicates a significant difference from negative control (sterile 0.1% Tween 80), *p* ≤ 0.05. Striped horizontal lines separate individual experiments.

Isolate	Taxon	Maize Variety	Emergence Success [n]	Emergence Time [d]	Root Length [cm]	Shoot Length [cm]	Total Plant Length [cm]	Plant Dry Weight [g]
**Control**		Chapalu	**4.67 ± 0.33**	4.33 ± 0.33	22.57 ± 1.15	32.87 ± 1.15	55.43 ± 2.45	0.30 ± 0.04
**1154**	*MB*	Chapalu	4.67 ± 0.33	4.13 ± 0.13	21.67 ± 1.63	30.90 ± 0.78	52.57 ± 2.28	0.29 ± 0.02
**1868**	*MB*	Chapalu	4.67 ± 0.33	4.33 ± 0.33	21.50 ± 0.15	29.60 ± 1.19	51.09 ± 1.21	0.28 ± 0.02
**2121**	*BB*	Chapalu	5.00 ± 0.00	4.40 ± 0.23	21.50 ± 1.10	32.27 ± 0.74	53.75 ± 1.73	0.30 ± 0.01
**Control**		Chapalu	**5.00 ± 0.00**	**4.27 ± 0.07**	**20.97 ± 0.45**	**29.97 ± 0.13**	**50.95 ± 0.37**	**0.27 ± 0.02**
**2631**	*MR*	Chapalu	4.67 ± 0.33	4.38 ± 0.32	21.23 ± 0.47	26.37 ± 0.43	47.58 ± 0.88	0.24 ± 0.01
**2632**	*MR*	Chapalu	4.33 ± 0.33	4.40 ± 0.31	21.40 ± 1.51	27.97 ± 0.65	49.37 ± 1.69	0.26 ± 0.01
**2245**	*MR*	Chapalu	5.00 ± 0.00	4.53 ± 0.07	18.80 ± 0.56	27.23 ± 1.43	46.04 ± 1.62	0.22 ± 0.02
**2246**	*MR*	Chapalu	4.67 ± 0.33	4.17 ± 0.17	18.50 ± 0.40	28.10 ± 0.81	46.56 ± 1.19	0.23 ± 0.01
**Control**		Chapalu	**5.00 ± 0.00**	**4.93 ± 0.07**	**21.23 ± 0.64**	**31.47 ± 0.83**	**52.70 ± 1.02**	**0.27 ± 0.01**
**2215**	*MR*	Chapalu	4.67 ± 0.33	4.43 ± 0.03	21.55 ± 0.75	29.30 ± 0.82	50.85 ± 0.08	0.25 ± 0.01
**2216**	*MR*	Chapalu	4.67 ± 0.33	4.57 ± 0.23	22.39 ± 0.55	29.82 ± 0.32	52.21 ± 0.73	0.25 ± 0.01
**2299**	*BB*	Chapalu	4.33 ± 0.33	4.40 ± 0.31	21.53 ± 0.48	26.43 ± 1.41	47.96 ± 1.81	0.21 ± 0.01
**2300**	*BB*	Chapalu	4.67 ± 0.33	4.40 ± 0.23	22.25 ± 1.02	27.40 ± 1.09	49.64 ± 2.10	0.22 ± 0.01
**Control**		Chapalu	**5.00 ± 0.00**	**4.13 ± 0.07**	**18.60 ± 0.95**	**29.57 ± 0.78**	**48.16 ± 1.71**	**0.25 ± 0.01**
**2635**	*MR*	Chapalu	4.67 ± 0.33	4.37 ± 0.09	21.40 ± 1.14	28.53 ± 0.96	49.94 ± 1.57	0.23 ± 0.03
**2637**	*MR*	Chapalu	4.67 ± 0.33	4.62 ± 0.50	19.93 ± 0.46	28.67 ± 1.08	48.59 ± 1.51	0.23 ± 0.02
**2641**	*MR*	Chapalu	5.00 ± 0.00	4.40 ± 0.31	19.57 ± 0.71	28.27 ± 1.07	47.81 ± 0.70	0.22 ± 0.02
**Control**		Chapalu	**4.67 ± 0.33**	**4.00 ± 0.00**	**16.30 ± 0.47**	**30.30 ± 1.30**	**46.61 ± 1.78**	**0.22 ± 0.01**
**2697**	*ND*	Chapalu	5.00 ± 0.00	4.07 ± 0.07	17.90 ± 0.23	27.27 ± 2.83	45.20 ± 2.70	0.23 ± 0.02
**2698**	*MR*	Chapalu	4.33 ± 0.67	4.44 ± 0.08	19.13 ± 0.99	27.60 ± 1.80	46.73 ± 2.17	0.21 ± 0.01
**2699**	*MR*	Chapalu	5.00 ± 0.00	4.20 ± 0.00	18.07 ± 0.61	31.27 ± 0.67	49.35 ± 1.20	0.22 ± 0.01
**2700**	*MR*	Chapalu	4.33 ± 0.33	4.53 ± 0.15	19.17 ± 0.24	33.30 ± 1.65	52.46 ± 1.83	0.26 ± 0.01
**Control**		Chapalu	**5.00 ± 0.00**	**4.07 ± 0.07**	**20.07 ± 1.28**	**30.33 ± 1.21**	**50.43 ± 2.28**	**0.22 ± 0.03**
**2298**	*BB*	Chapalu	4.33 ± 0.33	4.85 ± 0.52	20.80 ± 1.12	30.30 ± 0.66	51.09 ± 1.80	0.26 ± 0.02
**2243**	*MR*	Chapalu	5.00 ± 0.00	4.93 ± 0.13	15.60 ± 1.21	27.00 ± 0.95	42.59 ± 1.96	0.21 ± 0.01
**2636**	*MR*	Chapalu	5.00 ± 0.00	4.80 ± 0.23	16.77 ± 0.70	28.47 ± 0.38	45.26 ± 0.91	0.20 ± 0.02
**2642**	*MR*	Chapalu	4.33 ± 0.33	4.50 ± 0.25	17.20 ± 0.85	34.53 ± 0.32	51.72 ± 0.73	0.25 ± 0.01
**Control**		Chapalu	**4.67 ± 0.33**	**4.13 ± 0.07**	**20.70 ± 0.31**	**32.93 ± 1.88**	**53.61 ± 1.80**	**0.31 ± 0.05**
**2148**	*MR*	Chapalu	5.00 ± 0.00	4.27 ± 0.18	21.37 ± 0.45	35.77 ± 0.47	57.15 ± 0.50	0.32 ± 0.02
**2151**	*MR*	Chapalu	4.67 ± 0.33	4.57 ± 0.12	19.47 ± 0.58	36.77 ± 0.78	56.22 ± 0.50	0.32 ± 0.02
**2152**	*MR*	Chapalu	4.33 ± 0.33	4.28 ± 0.17	21.50 ± 0.64	35.67 ± 2.11	57.19 ± 2.76	0.31 ± 0.03
**2701**	*MR*	Chapalu	5.00 ± 0.00	4.47 ± 0.07	20.57 ± 0.62	33.60 ± 0.55	54.15 ± 0.38	0.33 ± 0.01
**Control**		Chapalu	**4.67 ± 0.33**	**4.20 ± 0.12**	**20.27 ± 0.26**	**34.70 ± 0.59**	**54.98 ± 0.83**	**0.32 ± 0.01**
**2703**	*MB*	Chapalu	4.33 ± 0.33	4.25 ± 0.25	24.43 ± 1.56	35.00 ± 1.59	59.44 ± 0.11	0.36 ± 0.01
**2009**	*MR*	Chapalu	5.00 ± 0.00	4.47 ± 0.18	20.70 ± 0.46	33.97 ± 2.72	54.65 ± 2.84	0.31 ± 0.04
**2010**	*MG*	Chapalu	5.00 ± 0.00	4.13 ± 0.13	20.40 ± 1.68	31.53 ± 1.82	51.94 ± 3.47	0.27 ± 0.04
**2011**	*MR*	Chapalu	5.00 ± 0.00	4.53 ± 0.13	20.93 ± 0.94	30.83 ± 1.43	51.81 ± 2.37	0.23 ± 0.00
**Control**		Chapalu	**4.67 ± 0.33**	**4.43 ± 0.07**	**13.03 ± 0.03**	**15.90 ± 1.46**	**28.91 ± 1.44**	**0.15 ± 0.01**
**2239**	*MR*	Chapalu	5.00 ± 0.00	4.59 ± 0.21	14.63 ± 1.76	18.90 ± 0.20	33.50 ± 1.56	0.19 ± 0.01
**2704**	*BB*	Chapalu	3.33 ± 0.33	5.00 ± 0.25	11.10 ± 0.50	18.00 ± 1.06	29.11 ± 1.30	0.17 ± 0.00
**2247**	*MG*	Chapalu	3.33 ± 0.33	4.81 ± 0.13	15.33 ± 1.06	18.80 ± 0.65	34.13 ± 1.61	0.19 ± 0.01
**2752**	*TA*	Chapalu	3.00 ± 0.58	5.29 ± 0.14	13.93 ± 0.73	18.40 ± 1.34	32.33 ± 2.03	0.19 ± 0.03
**2815**	*TB*	Chapalu	3.67 ± 0.33	4.71 ± 0.22	13.33 ± 1.48	18.20 ± 1.04	31.53 ± 2.35	0.20 ± 0.02
**2878**	*TH*	Chapalu	3.33 ± 0.33	4.44 ± 0.34	14.70 ± 1.51	18.73 ± 0.50	33.39 ± 1.62	0.17 ± 0.01
**2882**	*TA*	Chapalu	3.33 ± 0.33	4.84 ± 0.30	14.27 ± 1.08	20.00 ± 0.60	34.32 ± 1.11	0.21 ± 0.01
**2883**	*TG*	Chapalu	3.33 ± 0.33	4.41 ± 0.12	14.67 ± 1.21	19.47 ± 0.65	34.13 ± 1.83	0.17 ± 0.03
**Control**		Belokranjka	**9.00 ± 0.58**	**5.19 ± 0.05**	**23.87 ± 1.58**	**32.73 ± 0.83**	**56.58 ± 2.41**	**0.31 ± 0.03**
**2686**	*MR*	Belokranjka	10.00 ± 0.00	5.07 ± 0.20	24.50 ± 3.10	30.97 ± 0.58	55.48 ± 2.55	0.30 ± 0.01
**2687**	*MR*	Belokranjka	10.00 ± 0.00	5.33 ± 0.07	24.30 ± 2.31	30.70 ± 0.23	54.96 ± 2.22	0.30 ± 0.01
**2690**	*MB*	Belokranjka	9.33 ± 0.33	5.14 ± 0.07	23.23 ± 0.43	29.10 ± 0.40	52.35 ± 0.21	0.30 ± 0.01
**2692**	*MR*	Belokranjka	10.00 ± 0.00	5.20 ± 0.06	24.13 ± 2.70	30.90 ± 1.06	55.01 ± 2.82	0.29 ± 0.03
**Control**		Belokranjka	**9.67 ± 0.33**	**5.41 ± 0.11**	**25.17 ± 0.59**	**32.80 ± 1.36**	**57.95 ± 1.96**	**0.30 ± 0.02**
**2152**	*MR*	Belokranjka	10.00 ± 0.00	5.43 ± 0.07	28.00 ± 2.11	33.47 ± 0.23	61.46 ± 2.21	0.29 ± 0.01
**Control**		Belokranjka	**9.33 ± 0.33**	**5.11 ± 0.00**	**28.53 ± 0.87**	**26.87 ± 0.93**	**55.39 ± 0.82**	**0.27 ± 0.01**
**2146**	*MR*	Belokranjka	9.33 ± 0.33	5.22 ± 0.16	26.33 ± 1.87	30.00 ± 0.35	56.36 ± 1.75	0.35 ± 0.02
**2147**	*MR*	Belokranjka	10.00 ± 0.00	4.90 ± 0.21	24.83 ± 1.42	26.03 ± 0.43	50.84 ± 1.51	0.25 ± 0.02
**2251**	*MR*	Belokranjka	9.33 ± 0.33	5.31 ± 0.22	28.33 ± 3.80	28.00 ± 0.78	56.27 ± 4.42	0.27 ± 0.03
**2789**	*MR*	Belokranjka	9.67 ± 0.33	5.07 ± 0.14	27.23 ± 1.95	26.10 ± 1.01	53.37 ± 2.55	0.28 ± 0.02
**2793**	*MR*	Belokranjka	9.33 ± 0.67	4.93 ± 0.09	26.27 ± 2.03	27.47 ± 1.83	53.71 ± 1.97	0.30 ± 0.00
**2794**	*MR*	Belokranjka	8.67 ± 0.88	4.85 ± 0.03	29.03 ± 2.22	29.43 ± 0.50	58.47 ± 2.68	0.35 ± 0.03
**2795**	*MR*	Belokranjka	9.67 ± 0.33	5.27 ± 0.18	24.60 ± 2.01	29.10 ± 1.46	53.69 ± 0.85	0.27 ± 0.01
**Control**		Belokranjka	**9.67 ± 0.33**	**5.11 ± 0.21**	**19.60 ± 1.91**	**25.23 ± 1.12**	**44.81 ± 2.82**	**0.25 ± 0.01**
**2645**	*MR*	Belokranjka	9.67 ± 0.33	5.00 ± 0.06	23.57 ± 0.52	26.23 ± 1.36	49.76 ± 1.90	0.34 ± 0.06
**2691**	*MR*	Belokranjka	9.00 ± 0.00	5.26 ± 0.04	24.40 ± 3.21	25.50 ± 1.19	49.91 ± 4.36	0.30 ± 0.01
**2693**	*MR*	Belokranjka	10.00 ± 0.00	5.07 ± 0.09	23.93 ± 0.91	26.27 ± 0.78	50.19 ± 1.61	0.32 ± 0.04
**2790**	*MR*	Belokranjka	9.67 ± 0.33	5.34 ± 0.18	21.50 ± 0.49	27.37 ± 1.92	48.86 ± 2.42	0.31 ± 0.02
**Control**		Belokranjka	**8.33 ± 0.33**	**5.47 ± 0.16**	**20.63 ± 1.77**	**23.40 ± 1.08**	**44.05 ± 2.27**	**0.31 ± 0.03**
**2634**	*MR*	Belokranjka	9.33 ± 0.33	5.56 ± 0.20	21.20 ± 1.10	25.40 ± 0.71	46.57 ± 1.06	0.34 ± 0.06
**2214**	*MR*	Belokranjka	9.33 ± 0.33	5.25 ± 0.04	23.57 ± 0.73	27.07 ± 0.87	50.63 ± 0.22	0.33 ± 0.03
**2243**	*MR*	Belokranjka	9.00 ± 0.58	5.23 ± 0.07	23.90 ± 1.99	28.37 ± 1.06	52.25 ± 2.88	0.36 ± 0.00
**2702**	*MR*	Belokranjka	9.00 ± 0.00	5.07 ± 0.04	22.10 ± 2.80	27.07 ± 1.39	49.18 ± 3.64	0.32 ± 0.00
**2791**	*MR*	Belokranjka	9.33 ± 0.33	5.10 ± 0.10	25.33 ± 4.53	27.10 ± 1.61	52.42 ± 6.14	0.32 ± 0.03
**Control**		Belokranjka	**9.00 ± 0.58**	**4.91 ± 0.21**	**24.20 ± 0.38**	**28.30 ± 0.51**	**52.52 ± 0.21**	**0.31 ± 0.01**
**2250**	*MG*	Belokranjka	8.33 ± 0.88	5.13 ± 0.07	24.20 ± 2.08	26.77 ± 0.42	50.98 ± 1.81	0.26 ± 0.02
**2685**	*MR*	Belokranjka	9.00 ± 0.58	4.99 ± 0.11	22.50 ± 0.25	29.97 ± 1.39	52.45 ± 1.59	0.30 ± 0.02
**Control**		Belokranjka	**8.67 ± 0.33**	**5.23 ± 0.17**	**24.10 ± 0.75**	**26.63 ± 0.12**	**50.75 ± 0.67**	**0.29 ± 0.02**
**2640**	*MR*	Belokranjka	9.67 ± 0.33	5.17 ± 0.09	23.37 ± 2.00	26.10 ± 0.29	49.47 ± 1.89	0.28 ± 0.02
**2694**	*MR*	Belokranjka	9.67 ± 0.33	5.28 ± 0.32	24.87 ± 1.07	27.90 ± 0.66	52.77 ± 1.05	0.26 ± 0.00
**2695**	*MR*	Belokranjka	9.33 ± 0.33	5.11 ± 0.06	24.40 ± 2.94	26.57 ± 0.90	50.96 ± 3.77	0.28 ± 0.02
**2788**	*MR*	Belokranjka	8.67 ± 0.67	5.15 ± 0.27	23.87 ± 2.12	28.20 ± 1.33	52.07 ± 2.48	0.32 ± 0.02
**2792**	*MR*	Belokranjka	9.33 ± 0.33	5.57 ± 0.36	25.20 ± 3.54	28.10 ± 0.96	53.27 ± 4.46	0.29 ± 0.01
**2796**	*MR*	Belokranjka	8.67 ± 1.33	4.98 ± 0.11	21.90 ± 1.20	27.70 ± 2.06	49.62 ± 0.86	0.32 ± 0.02
**Control**		Belokranjka	**8.67 ± 0.88**	**4.67 ± 0.13**	**22.93 ± 1.71**	**28.00 ± 1.13**	**50.96 ± 2.47**	**0.29 ± 0.02**
**2688**	*MR*	Belokranjka	9.00 ± 0.58	5.10 ± 0.23	26.80 ± 0.68	28.97 ± 0.37	55.76 ± 0.48	0.36 ± 0.03
**Control**		Belokranjka	**8.33 ± 0.67**	**4.52 ± 0.24**	**15.53 ± 1.49**	**21.43 ± 0.88**	**36.97 ± 2.00**	**0.15 ± 0.01**
**2154**	*MR*	Belokranjka	7.67 ± 0.33	5.09 ± 0.25	14.90 ± 0.92	22.43 ± 0.78	37.35 ± 1.07	0.14 ± 0.01
**Control**		Belokranjka	**9.67 ± 0.33**	**4.14 ± 0.10**	**22.97 ± 0.84**	**26.53 ± 0.55**	**49.54 ± 1.18**	**0.23 ± 0.01**
**2150**	*MR*	Belokranjka	10.00 ± 0.00	4.47 ± 0.37	23.13 ± 1.39	27.07 ± 1.20	50.23 ± 2.56	0.26 ± 0.01
**2240**	*MR*	Belokranjka	9.67 ± 0.33	4.28 ± 0.09	24.07 ± 0.77	27.53 ± 1.78	51.60 ± 2.41	0.25 ± 0.02

Note: *MB*: *Metarhizium brunneum*; *MR*: *Metarhizium robertsii*; *MG*: *Metarhizium guizhouense*; *BB*: *Beauveria bassiana*; *TA*: *Trichoderma atroviride*; *TB*: *Trichoderma brevicompactum*; *TG*: *Trichoderma gamsii*; *TH*: *Trichoderma harzianum*; *ND*: No data.

**Table 3:** Growth stimulating effects of selected fungal isolates on maize (Chapalu variety) grown in sand with/without fertilizer for 21 days. Data presented are the mean values ± SE (n = 15 (3 replicates with 5 seeds each)). Green fill indicates isolates with significant growth promoting properties, red fill indicates isolates with growth inhibitory properties and grey fill indicates a significant difference from negative control (sterile 0.1% Tween 80), *p* ≤ 0.05.

		Emergence Success [n]		Emergence Time [d]		Root Length [cm]		Shoot Length [cm]		Total Plant Length [cm]		Plant Dry Weight [g]	
Isolate	Taxon	No Fertilizer	Fertilizer	No Fertilizer	Fertilizer	No Fertilizer	Fertilizer	No Fertilizer	Fertilizer	No Fertilizer	Fertilizer	No Fertilizer	Fertilizer
**Control**		**5.00 ± 0.00**	**4.33 ± 0.67**	**4.25 ± 0.00**	**4.42 ± 0.21**	**18.90 ± 0.00**	**16.27 ± 0.58**	**14.10 ± 0.00**	**16.77 ± 1.30**	**32.93 ± 0.00**	**33.05 ± 1.72**	**0.21 ± 0.00**	**0.27 ± 0.02**
**1154**	*MB*	5.00 ± 0.00	4.67 ± 0.33	4.60 ± 0.12	4.83 ± 0.34	16.23 ± 1.42	14.13 ± 0.74	12.93 ± 0.72	16.40 ± 0.46	29.11 ± 1.18	30.53 ± 1.00	0.25 ± 0.02	0.42 ± 0.04
**1868**	*MB*	4.67 ± 0.33	5.00 ± 0.00	4.33 ± 0.29	4.53 ± 0.07	17.90 ± 0.70	16.73 ± 0.72	13.50 ± 0.06	16.77 ± 0.81	31.38 ± 0.77	33.49 ± 0.40	0.24 ± 0.01	0.38 ± 0.05
**2121**	*BB*	5.00 ± 0.00	4.67 ± 0.33	4.72 ± 0.17	5.02 ± 0.13	15.53 ± 0.87	15.27 ± 0.23	15.07 ± 0.74	18.40 ± 0.59	30.65 ± 1.24	33.68 ± 0.76	0.30 ± 0.00	0.41 ± 0.09
**Control**		**5.00 ± 0.00**	**4.67 ± 0.33**	**4.67 ± 0.13**	**5.17 ± 0.20**	**14.63 ± 0.90**	**15.43 ± 0.92**	**13.40 ± 2.15**	**18.70 ± 1.36**	**28.04 ± 2.92**	**34.15 ± 2.29**	**0.28 ± 0.03**	**0.30 ± 0.02**
**2631**	*MR*	4.67 ± 0.33	5.00 ± 0.00	4.63 ± 0.09	4.40 ± 0.12	20.03 ± 0.96	18.77 ± 0.19	13.80 ± 0.65	17.47 ± 0.50	33.84 ± 1.20	36.23 ± 0.57	0.30 ± 0.01	0.31 ± 0.04
**2632**	*MR*	4.33 ± 0.67	5.00 ± 0.00	4.42 ± 0.14	4.53 ± 0.07	17.47 ± 0.99	19.67 ± 1.62	12.87 ± 0.43	17.50 ± 0.86	30.38 ± 1.32	37.17 ± 1.98	0.30 ± 0.03	0.27 ± 0.03
**2245**	*MR*	4.67 ± 0.33	5.00 ± 0.00	5.37 ± 0.63	4.53 ± 0.24	18.63 ± 0.58	19.33 ± 1.43	14.07 ± 0.73	17.83 ± 0.47	32.72 ± 0.99	37.16 ± 1.35	0.23 ± 0.02	0.27 ± 0.02
**2246**	*MR*	5.00 ± 0.00	4.67 ± 0.33	4.67 ± 0.24	5.15 ± 0.18	18.13 ± 0.64	19.33 ± 1.83	12.37 ± 0.37	18.67 ± 0.76	30.49 ± 1.00	37.99 ± 2.43	0.26 ± 0.03	0.30 ± 0.02
**Control**		**5.00 ± 0.00**	**5.00 ± 0.00**	**5.53 ± 0.37**	**5.47 ± 0.29**	**18.93 ± 0.92**	**17.95 ± 1.23**	**13.34 ± 1.26**	**18.80 ± 0.96**	**32.27 ± 1.92**	**36.75 ± 1.70**	**0.20 ± 0.02**	**0.23 ± 0.02**
**2215**	*MR*	5.00 ± 0.00	5.00 ± 0.00	5.87 ± 0.35	5.80 ± 0.12	17.15 ± 1.35	19.25 ± 1.35	13.19 ± 1.10	17.18 ± 0.33	30.33 ± 2.06	36.43 ± 1.40	0.19 ± 0.00	0.21 ± 0.01
**2216**	*MR*	4.67 ± 0.33	4.00 ± 0.58	5.68 ± 0.28	5.27 ± 0.27	18.92 ± 1.85	17.62 ± 1.43	13.50 ± 0.14	16.97 ± 0.97	32.42 ± 1.85	34.59 ± 1.36	0.25 ± 0.01	0.21 ± 0.01
**2299**	*BB*	4.67 ± 0.33	5.00 ± 0.00	5.93 ± 0.58	5.87 ± 0.29	12.94 ± 1.10	15.99 ± 1.95	14.98 ± 0.26	18.75 ± 0.36	27.92 ± 1.27	34.73 ± 1.60	0.18 ± 0.01	0.19 ± 0.01
**2300**	*BB*	4.67 ± 0.33	4.67 ± 0.33	5.18 ± 0.32	5.98 ± 0.21	19.94 ± 1.20	14.48 ± 0.74	15.33 ± 0.42	19.26 ± 0.22	35.26 ± 1.58	33.74 ± 0.58	0.23 ± 0.00	0.25 ± 0.02
**Control**		**5.00 ± 0.00**	**4.33 ± 0.67**	**5.27 ± 0.29**	**5.22 ± 0.51**	**19.67 ± 1.56**	**17.50 ± 1.53**	**13.97 ± 0.58**	**18.40 ± 0.57**	**33.63 ± 0.98**	**35.90 ± 1.97**	**0.19 ± 0.01**	**0.20 ± 0.02**
**2635**	*MR*	4.67 ± 0.33	4.67 ± 0.33	5.97 ± 0.27	6.13 ± 0.77	18.20 ± 0.90	16.30 ± 1.40	13.47 ± 1.22	19.30 ± 1.05	31.68 ± 1.18	35.62 ± 2.23	0.18 ± 0.01	0.21 ± 0.00
**2637**	*MR*	4.33 ± 0.33	4.67 ± 0.33	5.68 ± 0.09	5.65 ± 0.13	17.00 ± 0.75	19.60 ± 1.04	14.23 ± 0.38	19.63 ± 0.26	31.25 ± 1.14	39.23 ± 1.25	0.17 ± 0.00	0.22 ± 0.03
**2641**	*MR*	5.00 ± 0.00	5.00 ± 0.00	4.93 ± 0.07	5.07 ± 0.13	17.40 ± 0.61	18.73 ± 2.03	14.03 ± 0.42	20.07 ± 0.61	31.42 ± 0.61	38.77 ± 2.58	0.20 ± 0.01	0.21 ± 0.01
**Control**		**5.00 ± 0.00**	**4.67 ± 0.33**	**4.93 ± 0.07**	**5.07 ± 0.07**	**19.73 ± 1.36**	**18.27 ± 1.92**	**15.90 ± 0.23**	**19.33 ± 0.44**	**35.61 ± 1.28**	**37.59 ± 2.35**	**0.42 ± 0.04**	**0.41 ± 0.02**
**2697**	*ND*	5.00 ± 0.00	5.00 ± 0.00	4.40 ± 0.12	4.87 ± 0.24	19.03 ± 1.68	16.77 ± 1.93	14.30 ± 1.21	20.00 ± 0.96	33.34 ± 2.80	36.75 ± 2.64	0.40 ± 0.03	0.46 ± 0.04
**2698**	*MR*	4.67 ± 0.33	5.00 ± 0.00	4.80 ± 0.12	5.20 ± 0.40	17.10 ± 1.12	16.50 ± 1.65	15.10 ± 0.45	18.17 ± 0.90	32.20 ± 1.39	34.69 ± 2.31	0.37 ± 0.03	0.45 ± 0.04
**2699**	*MR*	4.67 ± 0.33	5.00 ± 0.00	5.42 ± 0.42	5.20 ± 0.23	19.87 ± 2.63	21.47 ± 2.86	14.67 ± 1.28	21.13 ± 0.78	34.51 ± 3.89	42.59 ± 2.23	0.37 ± 0.04	0.36 ± 0.02
**2700**	*MR*	4.67 ± 0.33	5.00 ± 0.00	5.08 ± 0.08	4.67 ± 0.24	19.43 ± 1.93	20.33 ± 0.83	15.33 ± 0.41	19.27 ± 0.48	34.73 ± 2.28	39.63 ± 0.99	0.34 ± 0.03	0.39 ± 0.01
**Control**		**5.00 ± 0.00**	**4.67 ± 0.33**	**5.33 ± 0.35**	**5.50 ± 0.06**	**21.07 ± 1.69**	**19.00 ± 1.30**	**15.67 ± 1.17**	**21.63 ± 0.85**	**36.75 ± 2.84**	**40.65 ± 0.84**	**0.39 ± 0.02**	**0.28 ± 0.07**
**2298**	*BB*	4.67 ± 0.33	4.67 ± 0.33	5.57 ± 0.12	4.92 ± 0.14	21.03 ± 1.07	22.33 ± 1.32	16.60 ± 0.61	21.63 ± 0.81	37.63 ± 1.58	43.97 ± 1.46	0.45 ± 0.03	0.44 ± 0.03
**2243**	*MR*	5.00 ± 0.00	5.00 ± 0.00	5.40 ± 0.23	5.20 ± 0.23	20.80 ± 1.22	21.27 ± 0.89	16.10 ± 0.30	21.27 ± 0.72	36.89 ± 1.52	42.51 ± 1.38	0.39 ± 0.04	0.39 ± 0.08
**2636**	*MR*	5.00 ± 0.00	5.00 ± 0.00	5.60 ± 0.35	5.13 ± 0.24	19.90 ± 1.35	20.87 ± 1.01	14.83 ± 1.03	20.20 ± 1.54	34.77 ± 2.35	41.04 ± 2.56	0.33 ± 0.04	0.37 ± 0.03
**2642**	*MR*	5.00 ± 0.00	4.67 ± 0.33	5.60 ± 0.12	5.20 ± 0.12	19.57 ± 1.12	21.07 ± 1.16	13.27 ± 1.03	18.93 ± 1.13	32.83 ± 2.10	39.99 ± 2.30	0.36 ± 0.01	0.32 ± 0.08
**Control**		**5.00 ± 0.00**	**5.00 ± 0.00**	**5.33 ± 0.47**	**4.87 ± 0.07**	**21.87 ± 0.84**	**18.77 ± 1.53**	**16.97 ± 0.38**	**21.03 ± 1.65**	**38.89 ± 1.14**	**39.79 ± 1.96**	**0.19 ± 0.01**	**0.21 ± 0.01**
**2148**	*MR*	5.00 ± 0.00	5.00 ± 0.00	4.40 ± 0.23	4.87 ± 0.07	17.67 ± 1.02	18.20 ± 1.51	14.63 ± 1.04	19.53 ± 1.02	32.28 ± 1.21	37.74 ± 1.92	0.20 ± 0.01	0.24 ± 0.01
**2151**	*MR*	4.67 ± 0.33	4.67 ± 0.33	4.83 ± 0.28	4.83 ± 0.34	20.80 ± 2.21	20.07 ± 0.70	15.87 ± 0.34	22.20 ± 0.42	36.65 ± 2.56	42.27 ± 0.38	0.21 ± 0.02	0.21 ± 0.00
**2152**	*MR*	4.33 ± 0.33	5.00 ± 0.00	5.15 ± 0.69	4.87 ± 0.13	20.50 ± 0.85	18.90 ± 0.87	16.07 ± 1.55	21.40 ± 0.85	36.59 ± 1.96	40.31 ± 0.08	0.19 ± 0.00	0.21 ± 0.02
**2701**	*MR*	5.00 ± 0.00	4.33 ± 0.33	4.80 ± 0.12	5.43 ± 0.70	20.90 ± 0.78	18.40 ± 0.87	15.50 ± 0.95	21.33 ± 0.38	36.37 ± 1.19	39.72 ± 1.23	0.20 ± 0.01	0.23 ± 0.00
**Control**		**4.33 ± 0.33**	**5.00 ± 0.00**	**4.90 ± 0.21**	**4.73 ± 0.24**	**19.47 ± 2.25**	**19.13 ± 0.58**	**14.83 ± 1.39**	**20.30 ± 1.30**	**34.27 ± 3.62**	**39.43 ± 1.83**	**0.21 ± 0.01**	**0.22 ± 0.00**
**2703**	*MB*	5.00 ± 0.00	5.00 ± 0.00	5.53 ± 0.27	5.87 ± 0.64	19.10 ± 1.66	21.13 ± 0.52	15.90 ± 0.32	18.40 ± 0.35	34.95 ± 1.35	39.51 ± 0.65	0.20 ± 0.00	0.23 ± 0.01
**2009**	*MR*	5.00 ± 0.00	3.67 ± 0.67	6.00 ± 0.53	5.87 ± 0.59	17.57 ± 0.62	17.97 ± 1.08	15.13 ± 1.82	22.87 ± 1.67	32.71 ± 2.32	40.83 ± 2.67	0.21 ± 0.02	0.23 ± 0.01
**2010**	*MG*	5.00 ± 0.00	5.00 ± 0.00	7.40 ± 0.90	6.20 ± 0.35	19.80 ± 1.59	16.37 ± 0.47	15.40 ± 1.56	21.93 ± 1.03	35.21 ± 0.57	38.30 ± 1.49	0.21 ± 0.00	0.21 ± 0.02
**2011**	*MR*	4.33 ± 0.33	4.67 ± 0.33	5.22 ± 0.46	5.60 ± 0.35	19.53 ± 0.78	19.40 ± 0.06	19.13 ± 0.42	22.97 ± 0.60	38.66 ± 1.16	42.40 ± 0.54	0.22 ± 0.02	0.23 ± 0.01

Note: *MB*: *Metarhizium brunneum*; *MR*: *Metarhizium robertsii*; *MG*: *Metarhizium guizhouense*; *BB*: *Beauveria bassiana*; *TA*: *Trichoderma atroviride*; *TB*: *Trichoderma brevicompactum*; *TG*: *Trichoderma gamsii*; *TH*: *Trichoderma harzianum*; ND: No data.

## Data Availability

Not applicable.

## Supplementary Materials

The following are available online at www.mdpi.com/2223-7747/10/11/2498/s1, Figure S1: Most virulent fungal isolates in pure culture (left) and on mycosed *Tenebrio molitor* larvae and adults (right). Isolates 1154 and 1868 are representatives of *Metarhizium brunneum*, isolates 2251 and 2637 representatives of *Metarhizium robertsii* and 2300 and 2121 are representatives of *Beauveria bassiana*. Figure S2: Virulence bioassay. Spore suspension in 50 mL Falcon tubes (left). After 15 s immersion in spore suspension, mealworm larvae were placed in a six-well plate (right).
