# Supplementary figures and images for: Testing Virulence of Different Species of Insect Associated Fungi against Yellow Mealworm (Coleoptera: Tenebrionidae) and Their Potential Growth Stimulation to Maize
Source: Plants (Basel). 2021 Nov 18;10(11):2498. doi: 10.3390/plants10112498 (PMC8623216; doi:10.3390/plants10112498)

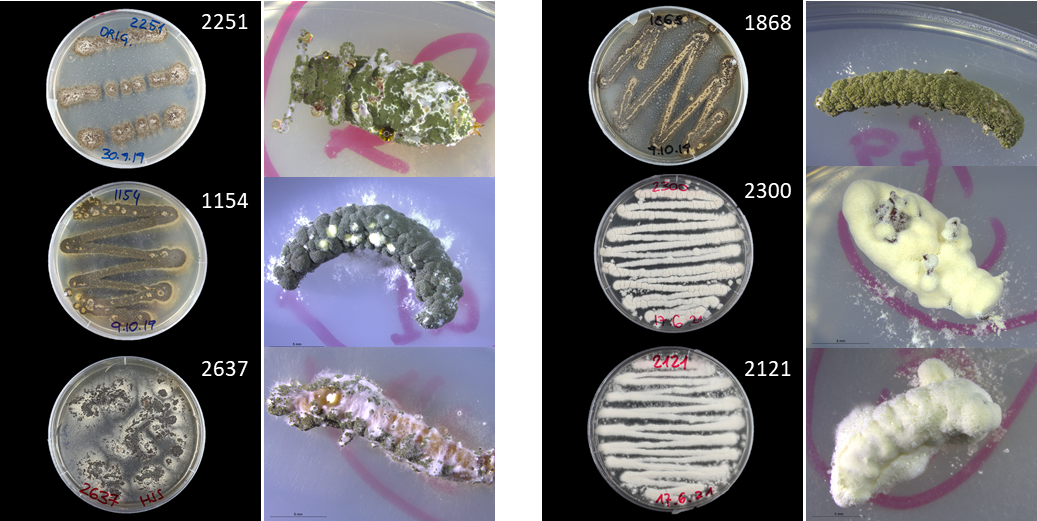

Supplement: Supplementary file 1 [file plants-10-02498-s001.zip › SuppFig1.png]

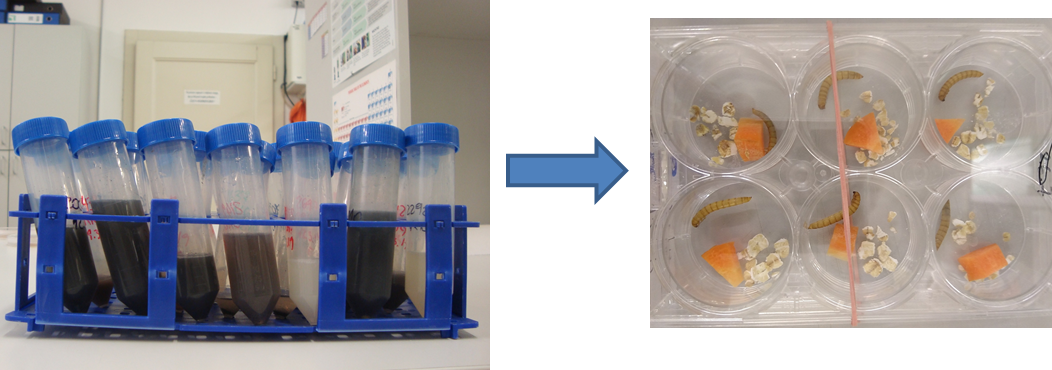

Supplement: Supplementary file 1 [file plants-10-02498-s001.zip › SuppFig2.png]
